# Fibrin binds to collagen and provides a bridge for αVβ3 integrin-dependent contraction of collagen gels

**DOI:** 10.1042/BJ20140201

**Published:** 2014-07-24

**Authors:** Vahid Reyhani, Pegah Seddigh, Bengt Guss, Renata Gustafsson, Lars Rask, Kristofer Rubin

**Affiliations:** *Department of Medical Biochemistry and Microbiology, Science for Life Laboratory, Uppsala University, BMC Box 582, SE-751 23 Uppsala, Sweden; †Department of Microbiology, BioCentrum, Swedish University of Agricultural Sciences, Box 7025, SE-750 07 Uppsala, Sweden; ‡Department of Experimental Medical Science, Lund University, 221 84 Lund, Sweden

**Keywords:** collagen type I, fibrin, gel contraction, interface matrix, protein binding, CNE, collagen-binding protein from *Steptococcus equi*, Col I, collagen type I, c-RGD, cyclic RGD, DMEM, Dulbecco’s modified Eagle’s medium, ECM, extracellular matrix, PAE, porcine aortic endothelial, PAR, protease-activated receptor, PBS-T, 0.05% Tween 20 in PBS, PDGF, platelet-derived growth factor, SPR, surface plasmon resonance

## Abstract

The functional significance of fibrin deposits typically seen in inflammatory lesions, carcinomas and in healing wounds is not fully understood. In the present study, we demonstrate that fibrinogen/fibrin specifically bound to native Col I (collagen type I) and used the Col I fibre network as a base to provide a functional interface matrix that connects cells to the Col I fibres through αVβ3 integrins. This allowed murine myoblast C2C12 cells to contract the collagenous composite gel via αVβ3 integrin. We show that fibrinogen specifically bound to immobilized native Col I at the site known to bind matrix metalloproteinase-1, discoidin domain receptor-2 and fibronectin, and that binding had no effect on Col I fibrillation. A specific competitive inhibitor blocking the Col-I-binding site for fibrinogen abolished the organization of fibrin into discernable fibrils, as well as the C2C12-mediated contraction of Col I gels. Our data show that fibrin can function as a linkage protein between Col I fibres and cells, and suggest that fibrin at inflammatory sites indirectly connects αVβ3 integrins to Col I fibres and thereby promotes cell-mediated contraction of collagenous tissue structures.

## Abstract 2

The putative functions of extravascular fibrin in pathologies are poorly characterized. We show that fibrinogen binds specifically to a defined protein-binding site of the native collagen. Through this binding, fibrin provides an interface matrix allowing αVβ3 integrin-mediated collagen gel contraction.

## INTRODUCTION

Fibrinogen is a 340 kDa fibrous glycoprotein consisting of two chains each of Aα-, Bβ- and γ-chains linked together by disulfide bonds. It is synthesized in the liver and circulates at a concentration of 1.5–3 g/l of plasma [1,2]. Fibrinogen is cleaved by thrombin as the final step in the coagulation cascade and is converted into fibrin monomers that form proto-fibrils, which mature to fibres. Stabilization of the fibres occurs by formation of intermolecular cross-links through the action of the transglutaminase Factor XIIIa [1,2]. Factor XIIIa also catalyses the formation of cross-links between fibrin and Col I (collagen type I), fibronectin and von Willebrand factor [2–4].

Extravascular fibrin is deposited in inflammatory lesions, carcinoma and healing wounds [5–8]. In healing wounds, the extravascular fibrin network forms a provisional matrix that serves as a substrate for cells such as fibroblasts [9,10] and endothelial cells [11] to migrate into the wounded area during the repair process. Fibrinogen is an acute-phase protein that, in addition to its essential role in haemostasis [12–14], promotes non-adaptive immune responses by activating specific signalling cascades in macrophages, at least in part mediated by αMβ2 [15,16]. Even though the pro-inflammatory effects of fibrin and its degradation products have been widely studied, the potential effects of fibrin on the ECM (extracellular matrix) at inflammatory sites and its putative effects on the contractile phenotype of fibroblasts are yet to be investigated.

Fibrinogen interacts with several cell-surface receptors expressed by different cell types. Human fibrinogen contains two RGD sites in the Aα-chain: Aα^95–98^ (RGDF) and Aα^572–575^ (RGDS) [17]. The platelet αIIbβ3 integrin recognizes Aα^572–575^ and a non-RGD decapeptide sequence of the γ-chain, γ^400–411^ (HHLGGAKQAGDV), in human fibrinogen [18]. The Aα^572–575^ (RGDS) site is recognized by the αVβ3 integrin expressed in fibroblasts and promotes attachment, but not spreading, of these cells [19]. Additional binding sites for αVβ3 are present in the human fibrinogen γ-chain: G^190^WTVFQKRLDGSV^202^ and G^346^VYYQGGTYSKAS^358^ [20]. Conversion of fibrinogen into fibrin exposes a cryptic leucocyte β2 integrin-binding site located in the γ-chain (γ^377–395^) [21]. Fibrinogen/fibrin also specifically associates with the ECM proteins thrombospondin, fibulin-1 and decorin, as well as the plasma proteins albumin, plasma fibronectin and von Willebrand factor [2,4,22].

Cells of different origin that are cultured in 3D-reconstituted collagen lattices contract the matrix by a process typically requiring hours to days (reviewed in [23]). Cellular mechanoforces are exerted on the fibril structure of the ECM, which results in contraction of the matrix. Several ECM proteins bind to collagen fibres and to cells via the αVβ3 integrin, and these proteins include vitronectin [24,25], periostin [26,27] and fibronectin [28,29]. We hypothesized that the deposited fibrin in inflamed lesions binds to collagen fibres and functionally links the fibres to cellular αVβ3 integrins, thereby allowing cell-mediated tissue contraction. To investigate these hypotheses, we took advantage of an *in vitro* model in which αVβ3-dependent cells were cultured in composite gels of fibrin and Col I.

## EXPERIMENTAL

### Cells

The murine C2C12 myoblast cell line was kindly provided by Dr Anna Starzinski-Powitz (Goethe-Universität, Frankfurt am Main, Germany). These cells lack functional collagen-binding β1 integrins. C2C12 cells stably transfected with the human α2 integrin subunit, denoted C2C12-α2β1 cells, have been described previously [30]. PAE (porcine aortic endothelial) cells were kindly donated by Dr Carl-Henrik Heldin (Ludwig Institute for Cancer Research, Uppsala, Sweden). The C2C12 and C2C12-α2β1 cells were cultured in DMEM (Dulbecco's modified Eagle's medium) with GlutaMAX™, whereas Ham's F12 medium (Gibco-BRL Life Technologies) was used for PAE cells. Cell culture medium was supplemented with 10% FBS (Biowest) and 50 μg/ml gentamicin (Gibco). Cells were cultured at 37°C and 5% CO_2_.

### Reagents

Human plasma fibrinogen and human thrombin were obtained from Sigma–Aldrich. PureCol native bovine dermal Col I (3 mg/ml) was purchased from Advanced Biomatrix. In SPR (surface plasmon resonance) experiments, mouse tail tendon Col I, extracted by 0.016 M acetic acid, was used in addition to PureCol. PDGF (platelet-derived growth factor)-BB was from Gibco-BRL Life Technologies. The c-RGD (cyclo-RGD) peptide cyclo-(Arg-Gly-Asp-D-Phe-Val), a highly selective inhibitor of the αVβ3 integrin [31], was from Bachem. The hamster anti-(mouse β3 integrin) antibody (HMβ3) was obtained from Life Technologies.

Streptococcal protein CNE (collagen-binding protein from *Steptococcus equi*) was produced and purified as described previously [32]. Biotin *N*-hydroxysuccinimide ester from Sigma–Aldrich was used to biotinylate proteins. Biotinylated proteins were applied on to PD-10 desalting columns (GE Healthcare) in PBS with 0.1% BSA (Sigma–Aldrich or Roche) with 0.02% sodium azide as a preservative. The avidin-conjugated alkaline phosphatase was from Vector Laboratories. Glutaraldehyde was from Agar Scientific, and cacodylate sodium salt from Sigma–Aldrich. Human fibronectin and vitronectin were purified from plasma as described previously [33,34]. Rabbit anti-(human fibrinogen) IgG was purchased from Biorbyt. Colloidal gold-conjugated Protein A and Aurion BSA-c™ (an acetylated and linearized form of BSA) were from Aurion. Triple helical collagen type II peptides II-1, II-37, II-44 and (GPP)_10_ were obtained from Profesor Richard Farndale (University of Cambridge, Cambridge, U.K.).

### Cell-mediated gel contraction

Cell-mediated gel contraction was performed essentially as described previously [35]. In brief, cells were trypsinized, suspended in serum-free DMEM and brought to 10^6^ cells/ml for all cell lines. The cell suspension was mixed on ice with gel solution at a 1:9 ratio. The gel solution for Col I gel contraction consisted of 5 vol. 2× DMEM, 1 vol. 0.2 M Hepes (pH 8.0) and 4 vol. Col I (3 mg/ml). For composite gel contraction, fibrinogen or fibronectin was also added to gels to a final concentration of 50 μg/ml. Fibrinogen solution in PBS was freshly made before the experiments by incubation at 37°C for 3–4 h. A portion (100 μl) of cell/gel mixture was added per well in a 96-well microtitre plate, blocked previously by incubation with 2% BSA in PBS to avoid binding of the gels to the plastic dish. Plates were incubated at 37°C for 1.5 h to allow gel polymerization. The gels were then floated with 100 μl of serum-free DMEM to provide free-floating gels for cells to contract freely. For fibrin/Col I composite gel contraction, thrombin was added to the floating solution to obtain a final concentration of 25×10^−4^ unit/ml. Inhibitors were added at the indicated concentrations in the gels and in the floatation solutions. Collagen gel contraction was measured at the indicated time points and recorded either as a decrease in gel area (using an inverted light microscope) or as weight loss (weighing the gels that had been fixed with 4% glutaraldehyde for 1 h at room temperature or overnight at 4°C).

### Scanning electron microscopy

SEM was performed on Col I and fibrin/Col I gels that were pre-pared, as described above, without and with cells. Gels were fixed with 2% glutaraldehyde in 0.15 M sodium cacodylate buffer (pH 7.4) overnight at 4°C. Samples were washed three times with 0.15 M sodium cacodylate buffer and subsequently dehydrated with an ethanol gradient, starting from 70%, to 90% to 100% (2×10 min for each ethanol concentration). Afterwards, samples were critical-point-dried, gold sputtered and analysed using a Leo 1530VP scanning electron microscope.

### Immunogold electron microscopy

Gels were fixed with 4% paraformaldehyde for 2 h at room temperature, followed by washing the samples with 0.2% Aurion BSA-c™ in PBS (6×10 min). To quench the unreacted aldehyde, samples were washed with 100 mM glycine in PBS for 10 min. Before primary antibody incubation, samples were blocked using Aurion blocking buffer for 2 h. Samples were incubated with the polyclonal rabbit anti-(human fibrinogen) antibody (1:500 dilution in the blocking solution) overnight at 4°C. Afterwards, gels were washed with 0.2% Aurion BSA-c™ in PBS (6×10 min), followed by overnight incubation with gold-conjugated Protein A (1:20 dilution) at 4°C. Samples were then fixed with SEM fix solution (2% glutaraldehyde in 0.15 M sodium cacodylate buffer, pH 7.4). Samples were prepared for SEM as described above and were subsequently analysed using back-scattered SEM in the Leo 1530VP scanning electron microscope. The specificity of the anti-(human fibrinogen) antibody was ascertained by immunohistochemical staining of experimental carcinoma sections. The antibody stained the collagenous stroma at low IgG concentrations, and the staining could be completely abolished by addition of low concentrations of soluble fibrinogen to the antibody solutions. Furthermore, the antibody did not stain normal mouse dermis.

### Solid-phase assay

Microtitre 96-well plates (SARSTEDT) were coated with native Col I (10 μg/ml), fibrinogen (10 μg/ml) or collagen type II peptides (10 μg/ml) and incubated overnight at 4°C. In order to avoid non-specific binding, plates were subsequently incubated with 2% BSA overnight at 4°C. Different concentrations of biotinylated protein were prepared in 0.5% BSA in Ca^2+^- and Mg^2+^-free PBS (pH 7.4) and added to the plates. After 2 h of incubation at 37°C to allow protein–protein interactions, plates were washed (three times) with PBS-T (0.05% Tween 20 in PBS). Afterwards, avidin-conjugated alkaline phosphatase (1:1000 dilution in PBS-T) was added to the plates for 2 h at room temperature. Plates were washed with PBS-T and developed with 0.6 mg/ml *p*-nitrophenyl phosphatase substrate in ethanolamine (Sigma–Aldrich) at 37°C until *A*_405_ was between 0.1 and 2 units.

### Collagen fibrillogenesis assay

The fibrillogenesis of pepsin-extracted Col I was monitored by the change in turbidity at 400 nm at 4-min intervals for up to 500 min. Four times concentrated buffer (80 mM Hepes and 0.6 M NaCl, pH 7.4), COMP (cartilage oligomeric matrix protein) and 0.012 M NaOH (in a volume equal to neutralize the collagen solution) were mixed, and water was added to a final volume of 250 μl. Col I in 0.012 M HCl was added, to a concentration of ~500 nM, within 1 min before initiation of the absorbance readings. Human fibrinogen was added to Col I to obtain the following concentrations: 130, 33 and 2 nM. The cuvettes were placed in a Beckman DU640 scanning spectrophotometer with a temperature-controlled six-place cuvette chamber equilibrated to 37°C. Fibrillogenesis of bovine dermal Col I (PureCol) was also monitored by a change in turbidity at 400 nm at 4-min intervals as described previously [34].

### Surface plasmon resonance

PureCol bovine dermal and mouse-tail tendon Col I were coupled to a BiaCore C1-chip using a Biosensor 2000 instrument (GE Healthcare), according to the protocol recommended by the manufacturer. Freshly dissolved fibrinogen in PBS that had been passed through a 0.2 mm filter was passed over the chip for 180 min, followed by a wash-out cycle for 400 min.

## RESULTS

### Fibrin induced αVβ3-dependent C2C12-mediated collagen gel contraction

**Figure 1:**
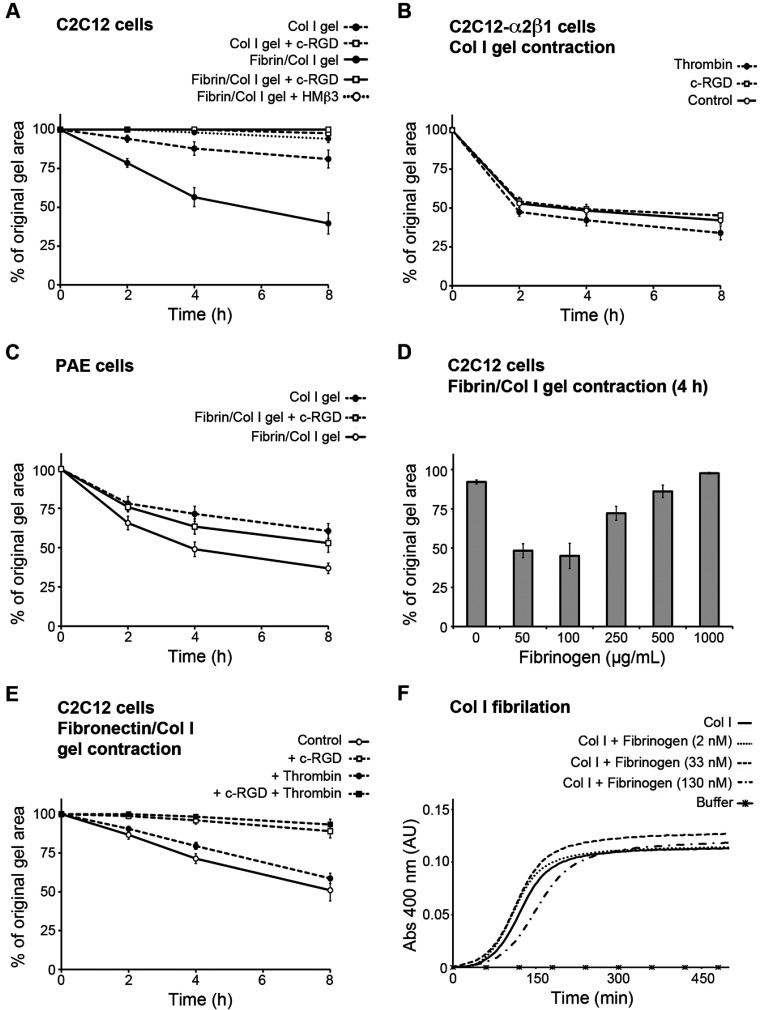
Fibrin triggers αVβ3 integrin-dependent cell-mediated contraction of collagen gels (**A**) Murine myoblast C2C12 cells that lack collagen-binding β1 integrins effectively contracted fibrin/Col I gels (Fib/Col I gel), but only marginally contracted pure Col I gels. Two selective αVβ3 integrin inhibitors, c-RGD (20 nM) and the anti-(mouse integrin-β3) antibody HMβ3 (10 μg/ml), abolished contraction. (**B**) C2C12 cells with forced expression of the α2β1 integrin (C2C12-α2β1 cells) contracted pure Col I gels to the same extent regardless of addition of 20 nM c-RGD or thrombin. (**C**) PAE cells, which express collagen-binding β1 integrins and αVβ3, showed additional contraction in response to the presence of fibrin in Col I gels. c-RGD at 20 nM repressed the fibrin-induced enhanced contraction. (**D**) C2C12 cells contracted fibrin/Col I gels in the presence of 50 and 100 μg/ml fibrin, but not at higher concentrations such as 500 and 1000 μg/ml fibrin. The values are after 4 h of contraction. (**E**) Thrombin did not enhance contraction of fibronectin/Col I (Fn/Col I) gels by C2C12 cells, whereas addition of 20 nM cRGD abolished contraction. (**F**) Similar fibrillation rates of Col I were observed in the absence or presence of fibrinogen (at three different concentrations). Values are means of at least three independent experiments performed in tetraplicates and error bars are S.E.M. Abs, absorbance; AU, arbitrary unit.

To investigate the effect of fibrin on the ability of cells to contract Col I gels in an αVβ3 integrin-dependent fashion, we used the mouse muscle satellite C2C12 cell line. C2C12 cells lack functional binding β1 integrins but express the αVβ3 integrin. In the absence of exogenously added stimulators, such as PDGF-BB, C2C12 cells are not capable of adhesion to native Col I or contraction of reconstituted Col I gels [36]. Therefore C2C12-mediated Col I gel contraction can serve as an *in vitro* model to investigate integrin αVβ3-mediated Col I gel contraction. In response to the addition of 50 μg/ml fibrinogen (equal to ~150 nM) into Col I gels (1 mg/ml collagen; equal to 3.3 μM), the C2C12 cells significantly contracted the resulting fibrin/Col I composite gels compared with pure Col I gels (Figure 1A). The selective inhibitor of the αVβ3 integrin, c-RGD peptide, at a concentration of 20 nM, which specifically inhibits αVβ3 [31], completely blocked the effective contraction of fibrin/Col I composite gels (Figure 1A). Similar to c-RGD, addition of 10 μg/ml HMβ3 antibody (inhibitor of the β3 integrin subunit) also completely inhibited the contraction (Figure 1A). As a control for potential toxicity of the c-RGD, C2C12-α2β1 cells were treated with c-RGD in the collagen gel contraction assay. C2C12-α2β1 cells express functional α2β1 and hence they can effectively contract pure Col I gels in the absence of exogenous stimulators. Addition of the same concentration of c-RGD had no effect on C2C12-α2β1-mediated Col I gel contraction, excluding the cytotoxicity of the peptide in the applied concentration (Figure 1B). In order to investigate whether fibrin-induced composite gel contraction could be observed with other cell lines, PAE cells were used both in composite gels and pure Col I gels. These cells express αVβ3 integrins, as well as collagen-binding β1 integrins. In presence of fibrin (50 μg/ml), PAE cells showed an enhanced gel contraction compared with contraction of pure Col I gels. Treatment of these cells with 20 nM c-RGD repressed the observed fibrin-enhanced gel contraction, emphasizing the dependency of fibrin-enhanced gel contraction on αVβ3 (Figure 1C).

### Fibrin concentration determines contractibility of collagen gels

The fibrin-triggered C2C12-mediated contraction of Col I gels was investigated further by addition of different concentrations of fibrinogen to Col I gels. The addition of fibrinogen at 50 and 100 μg/ml allowed for an effective C2C12-mediated Col I gel contraction, whereas higher concentrations, such as 500 and 1000 μg/ml, repressed gel contraction, presumably due to a prohibitively high stiffness (Figure 1D). Interestingly, fibril structure analysis of the composite gels consisting of a high concentration of fibrin (1000 μg/ml) showed a more dense fibril network with almost no empty spaces within the meshwork of fibrin fibres and Col I fibrils (see Figures 6I and 6J).

### Thrombin stimulation does not promote gel contraction by C2C12-α2β1 and C2C12 cells

Thrombin reportedly stimulates fibroblast-mediated collagen gel contraction through the activation of the PAR (protease-activated receptor)-1 expressed by the fibroblasts [37]. The role of thrombin in C2C12-mediated fibrin/Col I gel contraction was therefore investigated, as non-polymerized fibrinogen did not induce contraction and since thrombin was required to form fibrin in the composite gels during the contraction process. C2C12 cells express PAR-1, but non-detectable levels of PAR-2, -3 and -4 as studied by PCR. First, we investigated any potential effect of thrombin-induced activation of PARs on α2β1 integrin-mediated gel contraction. C2C12-α2β1 cells contracted pure Col I gels to the same degree in the absence and presence of thrombin (Figure 1B). At later time points (> 24 h), a slight additional contraction of pure Col I gels by C2C12-α2β1 cells was observed in response to thrombin. Secondly, the effect of thrombin on C2C12-mediated fibronectin/Col I gel contraction was investigated. Thrombin at the concentration used for fibrin/Col I gel contraction had no stimulatory effect on C2C12-mediated contraction of fibronectin/Col I gels (Figure 1E). Furthermore, thrombin was not able to induce C2C12-mediated contraction of pure Col I gels. Thirdly, PAR-1 antagonist and agonist peptides had no discernable effects either on C2C12 cell-mediated contraction of fibrin/Col I or C2C12-α2β1 cell-mediated contraction of pure Col I gels. These data do not fully exclude a stimulatory role of thrombin through PAR receptors on contraction, but emphasize the importance of thrombin-initiated polymerization of fibrinogen to fibrin that provides a secondary matrix dispersed within Col I fibre network, which in turn enables cell-mediated contraction of fibrin/Col I composite gels.

### Fibrinogen does not affect Col I fibrillation

To investigate whether addition of fibrinogen alters the fibril structure of the Col I gels, the Col I fibrillation was measured by turbidimetry in the presence of different concentrations of fibrinogen. No significant effects on collagen fibrillation were detected (Figure 1F).

### Fibrin involvement in C2C12 cell-mediated contraction of fibrin/Col I gels

**Figure 2:**
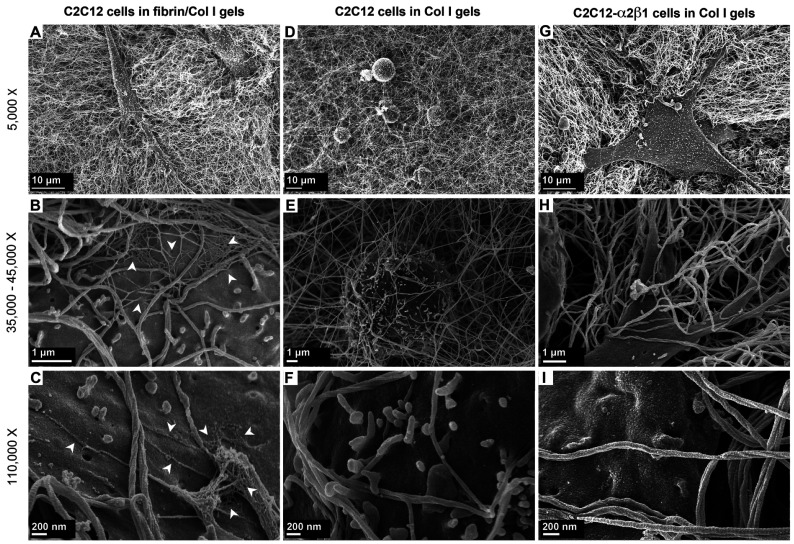
Fibrin forms a network within the Col I fibril network (**A**–**C**) The secondary network of fibrin within Col I fibrils was observed using SEM. Fibrin fibres were observed in associations with: (i) cell surfaces during gel contraction and (ii) Col I fibrils, suggesting the ‘bridging’ effect of fibrin between cells and Col I fibrils. The white arrows in (**B**) and (**C**) point to fibrin fibrils. After 12 h, the fibrin fibres were observed in closer associations with the cell surface (**C**) compared with at 4 h (**B**). (**D**–**F**) Representative micrographs of C2C12 cells in pure Col I gels. Cells were round and incapable of spreading after 4 h of culture. (**G**–**I**) Representative micrographs of C2C12-α2β1 cells in pure Col I gels. No secondary network, similar to fibrin fibrils (white arrows in **B** and **C**), was observed after either 4 h (**E**) or 12 h (**F**) of contraction.

**Figure 3:**
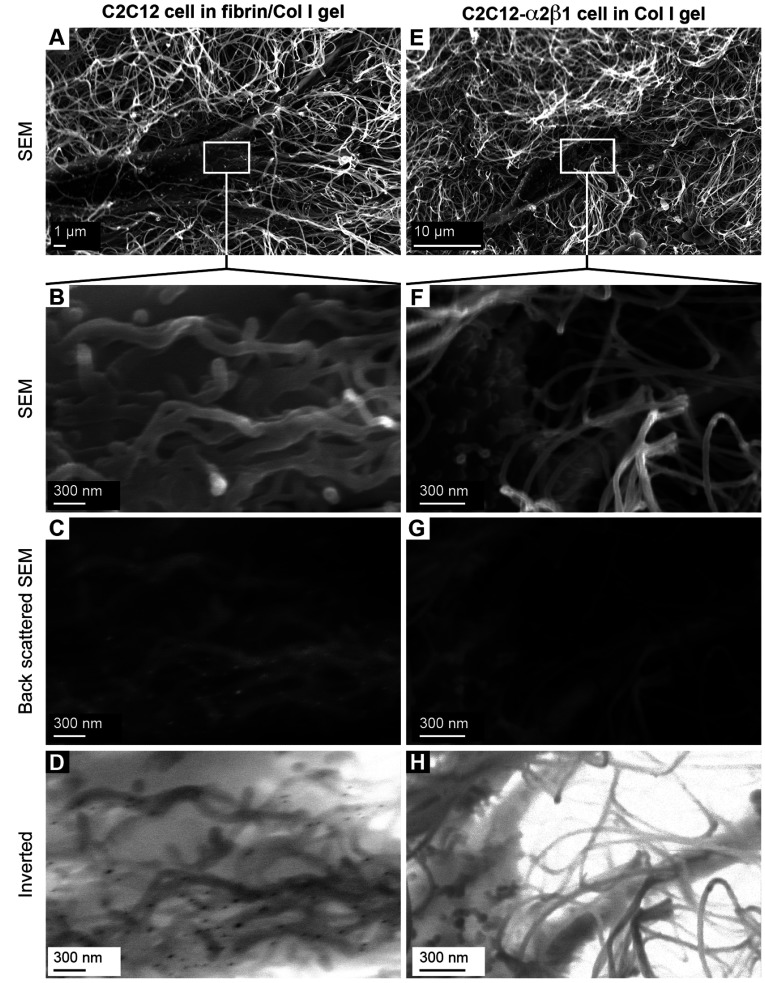
Immunogold staining of fibrinogen/fibrin in C2C12-mediated fibrin/collagen gel contraction (**A**–**D**) Fibrin was detected in close proximity to cell-membrane protrusions of C2C12 cells cultured in fibrin/Col I gels. The fibril structures around the cell surfaces were analysed using SEM, and back-scattered SEM was used to detect the gold particles (15 nm diameter). The gold particles are seen as white spots in (**C**) or black spots in the inverted image in (**D**). (**E**–**H**) Representative micrographs of C2C12-α2β1 cells in pure Col I gels that were treated and analysed identically with C2C12 cells in fibrin/Col I gels (**A**–**D**).

The morphology of C2C12 cells in the reconstituted fibrin/Col I gels was analysed using SEM (Figures 2A–2C). When collagen fibres had been formed, thrombin was added to gels allowing fibrin deposits to form. Fibrin formed a secondary fibrillar matrix interwoven with the collagen fibre network. Predominantly, fibrin formed an independent amorphous mesh between the Col I fibres (Figures 2B and see Figure 6B). However notably, in many instances, fibrin was directly associated with the collagen fibres (Figures 2B and 2C). Even at this low concentration, fibrin formed an amorphous meshwork that most often was not directly associated with the Col I fibres. Even though both collagen and fibrin fibres were observed in association with the cell surface, only fibrin fibres were found in tighter associations with the cell surface during the later time periods, as shown in Figure 2(C) (12 h contraction) compared with Figures 2(B) (4 h contraction). For comparison, C2C12 cells in pure Col I gels were studied. In pure Col I gels, the C2C12 cells were not capable of spreading (Figures 2D–2F). The C2C12-α2β1 cells were also studied in pure Col I gels (Figures 2G–2I). In the Col I gels, no fibril structure similar to the fibrin network (observed in composite gels, as in Figures 2A–2C) was found (Figures 2D–2I). The presence of fibrin in the composite gels was confirmed by immunogold staining of the fibrin/Col I gels. The signal from gold-conjugated Protein A, which was used to detect rabbit anti-fibrinogen IgG, was observed at the junctions of cell protrusions with fibrils of the composite gels (Figures 3A–3D). The C2C12-α2β1 cells in Col I gel samples were treated and analysed in the same way, but no gold particle stain was observed (Figures 3E–3H).

### Fibrinogen binds to native Col I

**Figure 4:**
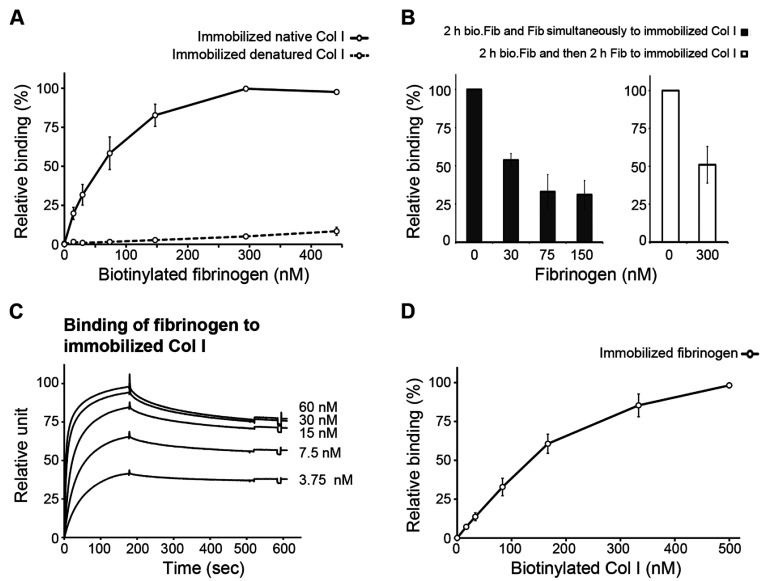
Fibrinogen binds to native Col I (**A**) Biotinylated fibrinogen bound to plates coated with 10 μg/ml native Col I, but not denatured Col I. Plates were incubated for 2 h at 37°C and bound fibrinogen was detected as described in the Experimental section. (**B**) Addition of non-biotinylated fibrinogen competed with the binding of biotinylated fibrinogen (bio.Fib) (~90 nM) to immobilized Col I (left-hand panel). To investigate the reversibility of the binding reaction, bound biotin-labelled fibrinogen was allowed to bind for 2 h and then the plates were washed and incubated for another 2 h with 300 nM unlabelled fibrinogen (right-hand panel). Remaining bound biotin-labelled fibrinogen was detected as described in the Experimental section. Values are expressed as a percentage of the binding of ~90 nM biotinylated fibrinogen to immobilized Col I. (**C**) Using SPR, maximal binding of fibrinogen to immobilized Col I was detected at a fibrinogen concentration of approximately 10 nM. The on-rate was high, but the off-rates during wash-out were slow and incomplete within the investigated time period. (**D**) Biotinylated Col I bound to immobilized fibrinogen in a concentration-dependent manner. Values are the means from at least three independent experiments that were performed in triplicate, and the error bars are S.E.M.

The possibility of a direct interaction between fibrinogen/fibrin and Col I was raised based on the EM analysis of fibrin/Col I composite gels, in which fibrin fibres were observed in a close association with the Col I fibrils. The potential binding of fibrinogen to Col I was investigated using a solid-phase assay. The biotinylated fibrinogen bound to immobilized native, but not to denatured, Col I in a saturable fashion using solid-phase assays performed for 2 h at 37°C (Figure 4A). Half-maximal binding was achieved at a fibrinogen concentration of ~70 nM. Addition of unlabelled fibrinogen competed with the biotinylated fibrinogen in binding to the immobilized Col I (Figure 4B). Bound biotinylated fibrinogen was displaced by unlabelled fibrinogen added at a 15-fold molar excess, suggesting an equilibrium type of binding (Figure 4B). Furthermore, investigations of the binding reaction using SPR showed that fibrinogen bound to immobilized Col I with a high on-rate (Figure 4C). Half-maximal binding in this system was achieved at a fibrinogen concentration of approximately 10 nM. The off-rates during wash-out were, however, slow and incomplete within the investigated time frame (Figure 4C). Biotinylated Col I on the other hand bound to immobilized fibrinogen in the same solid-phase setting, i.e. at a temperature allowing formation of collagen microfibrils (Figure 4D). Although no direct binding of fibrinogen to denatured collagen was detected in the solid-phase assays (Figure 4A), addition of increasing concentrations of fibronectin mediated binding of biotinylated fibrinogen to immobilized denatured Col I, which is in agreement with the reported ability of fibronectin to bind to fibrinogen [2] and collagen [38].

Attempts to inhibit binding of biotinylated fibrinogen to immobilized Col I by adding high amounts of soluble Col I to the reaction mixture and incubating at a low temperature (8°C) gave inconsistent results. Therefore we investigated whether fibrinogen interacts with soluble Col I, using turbidimetry in which no significant effects on collagen fibrillation were detected (Figure 1F), suggesting that fibrinogen does not interact effectively with soluble native Col I.

### Fibrinogen binds to the matrix-binding site of Col I fibres

**Figure 5:**
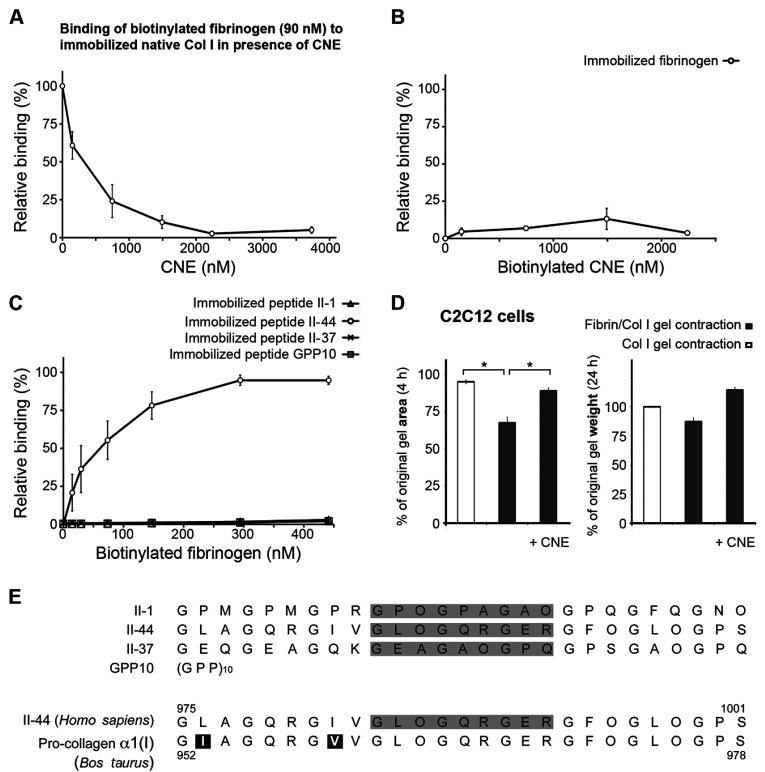
Fibrinogen binds to matrix-binding site of Col I fibres and this binding is essential for fibrin/Col I gel contraction (**A**) The collagen-binding protein CNE at 100 μg/ml completely inhibited the binding of biotinylated fibrinogen (~90 nM) to immobilized native Col I using a solid-phase assay. (**B**) No binding of biotinylated CNE to immobilized fibrinogen was detected (solid-phase assay). (**C**) Biotinylated fibrinogen only bound to immobilized collagen peptide II-44 from Toolkit II. Peptides II-1 and II-44 are ligands for CNE and peptides II-37 and (GPP)_10_ were used as controls. (**D**) C2C12 cells were allowed to contract either pure Col I gels or fibrin/Col I gels. Addition of 25 μg/ml CNE (~375 nM) abolished the fibrin-enhanced contraction of composite gels. Left-hand panel, values are relative to the original gel area after 4 h of contraction (*P<0.05). Right-hand panel, values are the relative weight loss of gels after 24 h of contraction by C2C12 cells. In all panels the values are the means from at least three independent experiments performed in at least triplicate, and the error bars are S.E.M. (**E**) The amino acid sequence of peptides II-1, II-44, II-37 and (GPP)_10_ from human collagen Toolkit II, as well as the region of human (*Homo sapiens*) pro-collagen α1(II) and bovine (*Bos taurus*) pro-collagen α1(I) that are covered by peptide II-44. Peptide II-44 has 93% homology with bovine pro-collagen α1(I).

To characterize further the binding of fibrinogen to Col I, the streptococcal protein CNE that specifically binds to fibronectin and to two sites in collagen type II (one at the N-terminus of the triple-helical part of collagen and one at a site known to bind matrix metalloproteinase-1 and discoidin domain receptor-2), was used [36,38–40]. CNE effectively inhibited the binding of fibrinogen to immobilized Col I in a dose-dependent manner (Figure 5A). Soluble biotinylated CNE did not bind to immobilized fibrinogen (Figure 5B). To investigate which of the two CNE-binding sites on collagen interacts with fibrinogen, synthetic native collagen peptides derived from the Toolkit collagen II library were used [39]. Biotinylated fibrinogen bound to immobilized peptide II-44, but not to immobilized peptides II-1 or II-37, nor to the control peptide (GPP)_10_ (Figure 5C). In summary, these findings show that fibrinogen specifically recognizes a site in the collagen triple helix and that it also specifically binds to immobilized native Col I.

### Fibrin/Col I binding is crucial for C2C12-mediated fibrin/Col I gel contraction

The streptococcal protein CNE effectively inhibits αVβ3-directed PDGF-BB-stimulated collagen gel contraction by C2C12 cells through specifically blocking the binding of cell-produced fibronectin to the Col I fibres [36]. To investigate whether a fibrin network can trigger contraction on its own or if it has to be anchored to the collagen lattice in order for cells to contract the gel, CNE was used in C2C12-mediated fibrin/Col I gel contraction. Strikingly, the fibrin-enhanced gel contraction was abolished in the presence of ~375 nM CNE. The results are presented as a percentage of the original gel area after 4 h or a percentage of the original gel weight after 24 h (Figure 5D).

### Fibrin/Col I binding stabilizes the fibrin network formed within the Col I fibre lattice

**Figure 6:**
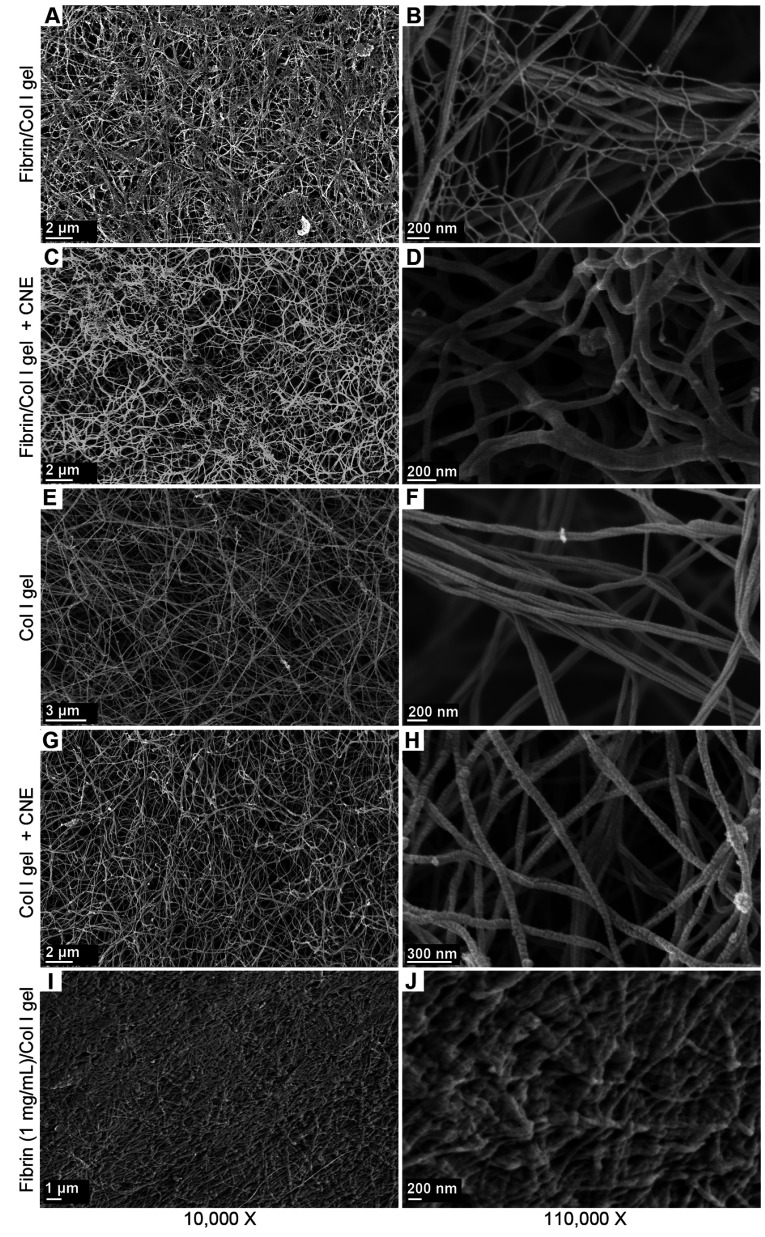
Fibrin binds to the matrix-binding site of Col I fibres and this binding stabilizes the secondary network of fibrin (**A** and **B**) Representative SEM images of fibrin (50 μg/ml)/Col I (1 mg/ml) gels. Thin fibres of fibrin were interwoven with the thicker Col I fibres forming a secondary network. (**C** and **D**) CNE, a streptococcal protein that inhibits the binding of fibrinogen to Col I, at a concentration of ~350 nM abolished the association of thin fibrin fibrils with the Col I fibre network. (**E** and **F**) Representative micrographs of pure Col I gels. (**G** and **H**) CNE (350 nM) had no overt effect on the fibre network structure of pure Col I gels. (**I** and **J**) Representative micrographs of fibrin/Col I composite gels consisting of a high concentration of fibrin (1 mg/ml) in Col I (1 mg/ml).

The morphology of reconstituted fibrin/Col I gels formed in the absence or presence of the streptococcal protein CNE was analysed using SEM (Figures 6A–6H). Even at a low concentration, such as 50 μg/ml, fibrin formed a secondary fibrillar matrix within the collagen fibre network, which at many instances was directly associated with the Col I fibres (Figures 6A and 6B). Addition of CNE (~375 nM), which blocked the binding of fibrinogen to Col I (Figure 5A), during the formation of the fibrin/Col I gels resulted in the disappearance of the fibrin network (Figures 6C and 6D). The fibril structure of collagen gels formed in the absence or presence of CNE was also analysed (Figures 6E–6H). Since CNE exclusively binds the collagenous component in the mixture (Figure 5B), these results strongly suggest that fibrinogen/fibrin has to directly interact with the Col I fibres for a fibrin fibrillar network to form. Thus our findings suggest that fibrin, similarly to fibrinogen, bound to native Col I.

Addition of higher concentrations of fibrin, such as 1000 μg/ml, to the composite gels of fibrin/Col I significantly increased the density of the fibril network and filled up of all the spaces between fibres, which were observed in the composite gels with 50 μg/ml fibrin (Figures 6I and 6J).

**Figure 7:**
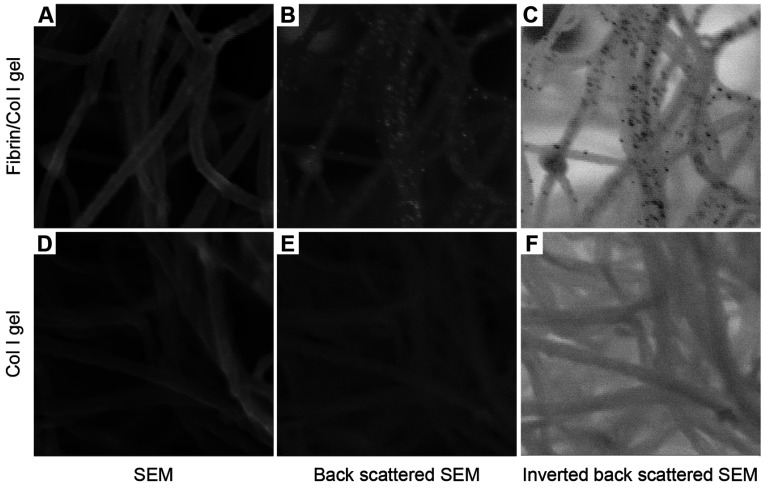
Immunogold staining of the fibrinogen/fibrin in fibrin/Col I gels (**A**–**C**) The presence of fibrinogen/fibrin in composite gels of fibrin/Col I was confirmed using immunogold SEM, as described in the Experimental section. Anti-fibrinogen IgG bound to fibrin/fibrinogen was detected by gold-conjugated Protein A (15 nm gold particles). Gold particles were visualized using back-scattered SEM (white spots in **B** or black spots in the inverted image in **C**). (**D**–**F**) Representative micrographs of pure Col I gels treated and analysed with the same procedure as used for immunogold staining of fibrin/Col I composite gels. No bound gold particles were detected in these gels.

The presence of fibrin in composite gels was confirmed by immunogold staining of the composite fibrin/collagen gels. Fibrin was observed only in the composite gels, but not in pure Col I gels that were treated identically (Figure 7). These results provide morphological support for a direct association of fibrin with collagen fibres.

## DISCUSSION

In the present study, we show that fibrinogen/fibrin directly binds to native Col I in the form of reconstituted collagen fibres or immobilized collagen on surfaces. The binding is specific to the extent that fibrinogen at nanomolar concentrations bound to immobilized native collagen, but not denatured collagen. The binding depends on a discrete binding site present in the native triple helix. The latter notion is based on the observation that the streptococcal protein CNE, which binds to two characterized sites along the collagen triple helix [36], effectively inhibited binding of fibrinogen to collagen and that fibrinogen specifically bound to one of these sites. These biochemical findings were strengthened by morphological investigations using SEM analyses that demonstrated frequent associations of thin fibrin fibrils to collagen fibres in reconstituted Col I gels containing low concentrations of fibrin. Analyses by immunogold SEM confirmed the presence of fibrinogen/fibrin directly associated with the collagen fibres. The specificity of this association is underscored by the observation that CNE, added during the formation of the composite gels, totally abolished a detectable fibrin network. This finding shows that fibrin, similarly to fibrinogen, directly bound Col I fibres and, furthermore, that the collagen network can serve as a template during formation of fibrin fibrils. Previous studies have pointed to a direct role for Factor XIIIa in the Ca^2+^-dependent binding of fibrin to collagen, resulting in the formation of covalent cross-links between the two fibrous proteins [3]. Our present results show an apparent binding of monomeric fibrinogen to immobilized Col I under Ca^2+^-free conditions and in the absence of thrombin or other plasma proteins, which demonstrates a direct molecular interaction between the two proteins.

CNE, which inhibited the fibrinogen–fibrin interactions with Col I, binds peptides II-1 and II-44 [36] from the Toolkit II library of overlapping triple helical collagen type II peptides [39]. Each Toolkit peptide comprises 27 amino acids of which the central nine are unique to each peptide. The collagen site defined by peptide II-1 contains the N-terminal triple helical domain. Peptide II-44 covers a collagen site that binds matrix metalloproteinase-1 (collagenase) [40] and discoidin domain receptor-2 [39], which is the site that supports PDGF-BB-stimulated integrin αVβ3-mediated adhesion to native collagen, as well as collagen gel contraction, through binding of cell-produced fibronectin [36]. A peptide identical with the unique nine central amino acids of II-44 is present in the pro-collagen α1(I) chain at a site located in the same region of Col I as II-44 in collagen type II (Figure 5E). In previous experiments, we found that peptide II-1 inhibited fibrillogenesis of diluted solutions of collagen types I and II, whereas peptide II-44 lacked this effect [36]. Taken together with the present finding that fibrillogenesis of dilute Col I solutions was not affected by soluble fibrinogen, it is more likely that fibrinogen interacts with the peptide II-44. Indeed, in solid-phase assays, fibrinogen bound to immobilized peptide II-44, but not to peptides II-1, II-37 (random peptide from Toolkit II as a control) or to (GPP)_10_. It is an interesting possibility that fibrinogen/fibrin competes with proteins such as fibronectin and matrix metalloproteinase-1 for the same binding site on collagen fibres, i.e. the binding site defined by the peptide II-44; such a competition will attribute an ECM-modulating role of fibrin deposited in tissues during inflammation, carcinoma and healing wounds.

Fibrin fibrils, associated with collagen in composite gels, induced contraction of the gel by C2C12 and PAE cells in the absence of exogenous stimulators, such as PDGF-BB or FBS. C2C12 cells that lack collagen-binding β1 integrins are unable to initiate collagen gel contraction unless stimulated by PDGF-BB. This process occurs after a lag phase and is dependent on the production of fibronectin by the cells [36]. This fibronectin can bind to the collagen fibres and expose binding sites for the αVβ3 integrin, thereby allowing the cells to contract the collagen network. CNE inhibits C2C12-mediated PDGF-BB-stimulated contraction of pure collagen gels, presumably by blocking the interaction of cell-produced fibronectin with the native collagen fibres [36]. The finding reported in the present study that CNE blocked fibrin-induced gel contraction by C2C12 cells emphasizes that the fibrin fibrils need to connect directly to the collagen network for contraction to occur. In accordance with previous findings [36], C2C12-α2β1 cells effectively contracted pure collagen gels in the absence of exogenous stimulators. Interestingly, the C2C12-α2β1-mediated Col I gel contraction was not sensitive to the presence of an inhibitor of αVβ3 integrin, c-RGD. Furthermore, PAE cells, which express collagen-binding β1 integrins, as well as αVβ3 integrins, showed increased contraction of Col I gels in response to the addition of fibrin to the gels. The additional contraction was abolished by the addition of c-RGD. This shows that fibrinogen/fibrin does not shroud collagen-binding sites for α2β1. Similar to α2β1, αVβ3 not only mediates physical contacts, but also induces signals that promote an effective and rapid contraction of collagen gels when presented with a dense enough substrate. Immunogold SEM revealed that C2C12 cells cultured in fibrin/Col I composite gels integrated at their cell surface thin fibrils that were labelled by anti-fibrinogen antibodies and thus could be identified as fibrin fibrils. It can, however, not be excluded that thin fibronectin fibrils are also present in these structures.

Fibrin is deposited in loose connective tissue structures during inflammatory reactions and in carcinoma [5–8]. Increased vascular leakage in affected lesions allows fibrinogen and pro-thrombin to enter tissues where thrombin can be generated, for example by pro-thrombinase activity in small lipid-containing vesicles secreted from activated macrophages [41]. Fibrin deposited in tissues can be pro-inflammatory and is continuously turned over, but accumulates in chronic conditions such as in rheumatoid arthritis, Duchenne muscular dystrophy and carcinoma [15,16,42,43].

In summary, we provide evidence of a direct and specific interaction between native Col I and fibrin/fibrinogen. Furthermore our results show that fibrin induces cell-mediated and αVβ3-dependent contraction of collagen gels. These findings suggest that fibrin deposition may aid during reinstatement of tissue homoeostasis and modulation of inflammation by triggering αVβ3-mediated tissue contraction.

## References

[1] Lord S. T. (2007). Fibrinogen and fibrin: scaffold proteins in hemostasis. Curr. Opin. Hematol..

[2] Weisel J. W. (2005). Fibrinogen and fibrin. Adv. Protein Chem..

[3] Duckert F., Nyman D. (1978). Factor XIII, fibrin and collagen. Suppl. Thromb. Haemost..

[4] Hada M., Kaminski M., Bockenstedt P., McDonagh J. (1986). Covalent crosslinking of von Willebrand factor to fibrin. Blood.

[5] Chu A. J. (2010). Blood coagulation as an intrinsic pathway for proinflammation: a mini review. Inflamm. Allergy Drug Targets.

[6] Colvin R. B., Johnson R. A., Mihm M. C., Dvorak H. F. (1973). Role of the clotting system in cell-mediated hypersensitivity. I. Fibrin deposition in delayed skin reactions in man. J. Exp. Med..

[7] Levi M., van der Poll T., Buller H. R. (2004). Bidirectional relation between inflammation and coagulation. Circulation.

[8] Schafer M., Werner S. (2008). Cancer as an overhealing wound: an old hypothesis revisited. Nat. Rev. Mol. Cell Biol..

[9] Brown L. F., Lanir N., McDonagh J., Tognazzi K., Dvorak A. M., Dvorak H. F. (1993). Fibroblast migration in fibrin gel matrices. Am. J. Pathol..

[10] Greiling D., Clark R. A. (1997). Fibronectin provides a conduit for fibroblast transmigration from collagenous stroma into fibrin clot provisional matrix. J. Cell Sci..

[11] Clark R. A., Tonnesen M. G., Gailit J., Cheresh D. A. (1996). Transient functional expression of αVβ3 on vascular cells during wound repair. Am. J. Pathol..

[12] Kannel W. B. (2005). Overview of hemostatic factors involved in atherosclerotic cardiovascular disease. Lipids.

[13] Mosesson M. W. (2005). Fibrinogen and fibrin structure and functions. J. Thromb. Haemost..

[14] Koenig W. (2003). Fibrin(ogen) in cardiovascular disease: an update. Thromb. Haemost..

[15] Jennewein C., Tran N., Paulus P., Ellinghaus P., Eble J. A., Zacharowski K. (2011). Novel aspects of fibrin(ogen) fragments during inflammation. Mol. Med..

[16] Davalos D., Akassoglou K. (2012). Fibrinogen as a key regulator of inflammation in disease. Semin. Immunopathol..

[17] Doolittle R. F., Watt K. W., Cottrell B. A., Strong D. D., Riley M. (1979). The amino acid sequence of the α-chain of human fibrinogen. Nature.

[18] Sanchez-Cortes J., Mrksich M. (2009). The platelet integrin αIIbβ3 binds to the RGD and AGD motifs in fibrinogen. Chem. Biol..

[19] Gailit J., Clarke C., Newman D., Tonnesen M. G., Mosesson M. W., Clark R. A. (1997). Human fibroblasts bind directly to fibrinogen at RGD sites through integrin αVβ3. Exp. Cell Res..

[20] Yokoyama K., Erickson H. P., Ikeda Y., Takada Y. (2000). Identification of amino acid sequences in fibrinogen γ-chain and tenascin C C-terminal domains critical for binding to integrin αVβ3. J. Biol. Chem..

[21] Ugarova T. P., Lishko V. K., Podolnikova N. P., Okumura N., Merkulov S. M., Yakubenko V. P., Yee V. C., Lord S. T., Haas T. A. (2003). Sequence γ377–395(P2), but not γ190–202(P1), is the binding site for the αMI-domain of integrin αMβ2 in the γ C-domain of fibrinogen. Biochemistry.

[22] Dugan T. A., Yang V. W., McQuillan D. J., Höök M. (2006). Decorin modulates fibrin assembly and structure. J. Biol. Chem..

[23] Grinnell F., Petroll W. M. (2010). Cell motility and mechanics in three-dimensional collagen matrices. Annu. Rev. Cell Dev. Biol..

[24] Gebb C., Hayman E. G., Engvall E., Ruoslahti E. (1986). Interaction of vitronectin with collagen. J. Biol. Chem..

[25] Pytela R., Pierschbacher M. D., Ruoslahti E. (1985). A 125/115-kDa cell surface receptor specific for vitronectin interacts with the arginine-glycine-aspartic acid adhesion sequence derived from fibronectin. Proc. Natl. Acad. Sci. U.S.A..

[26] Gillan L., Matei D., Fishman D. A., Gerbin C. S., Karlan B. Y., Chang D. D. (2002). Periostin secreted by epithelial ovarian carcinoma is a ligand for αVβ3 and αVβ5 integrins and promotes cell motility. Cancer Res..

[27] Norris R. A., Damon B., Mironov V., Kasyanov V., Ramamurthi A., Moreno-Rodriguez R., Trusk T., Potts J. D., Goodwin R. L., Davis J. (2007). Periostin regulates collagen fibrillogenesis and the biomechanical properties of connective tissues. J. Cell. Biochem..

[28] Engvall E., Ruoslahti E., Miller E. J. (1978). Affinity of fibronectin to collagens of different genetic types and to fibrinogen. J. Exp. Med..

[29] Yang J. T., Hynes R. O. (1996). Fibronectin receptor functions in embryonic cells deficient in α5β1 integrin can be replaced by αV integrins. Mol. Biol. Cell..

[30] Tiger C. F., Fougerousse F., Grundström G., Velling T., Gullberg D. (2001). α11β1 integrin is a receptor for interstitial collagens involved in cell migration and collagen reorganization on mesenchymal nonmuscle cells. Dev. Biol..

[31] Pfaff M., Tangemann K., Müller B., Gurrath M., Müller G., Kessler H., Timpl R., Engel J. (1994). Selective recognition of cyclic RGD peptides of NMR defined conformation by αIIbβ3, αVβ3, and α5β1 integrins. J. Biol. Chem..

[32] Lannergård J., Frykberg L., Guss B. (2003). CNE, a collagen-binding protein of *Streptococcus equi*. FEMS Microbiol. Lett..

[33] Miekka S. I., Ingham K. C., Menache D. (1982). Rapid methods for isolation of human plasma fibronectin. Thromb. Res..

[34] Hayashi M. (1994). Purification of vitronectin* from animal serum and plasma. J. Tissue Culture Method..

[35] Gullberg D., Tingström A., Thuresson A. C., Olsson L., Terracio L., Borg T. K., Rubin K. (1990). β1 integrin-mediated collagen gel contraction is stimulated by PDGF. Exp. Cell Res..

[36] van Wieringen T., Kalamajski S., Liden Å., Bihan D., Guss B., Heinegård D., Farndale R. W., Rubin K. (2010). The streptococcal collagen-binding protein CNE specifically interferes with αVβ3-mediated cellular interactions with triple helical collagen. J. Biol. Chem..

[37] Fang Q., Liu X., Abe S., Kobayashi T., Wang X. Q., Kohyama T., Hashimoto M., Wyatt T., Rennard S. I. (2004). Thrombin induces collagen gel contraction partially through PAR1 activation and PKC-ε. Eur. Respir. J..

[38] Erat M. C., Schwarz-Linek U., Pickford A. R., Farndale R. W., Campbell I. D., Vakonakis I. (2010). Implications for collagen binding from the crystallographic structure of fibronectin 6FnI1–2FnII7FnI. J. Biol. Chem..

[39] Farndale R. W., Lisman T., Bihan D., Hamaia S., Smerling C. S., Pugh N., Konitsiotis A., Leitinger B., de Groot P. G., Jarvis G. E., Raynal N. (2008). Cell-collagen interactions: the use of peptide Toolkits to investigate collagen-receptor interactions. Biochem. Soc. Trans..

[40] Manka S. W., Carafoli F., Visse R., Bihan D., Raynal N., Farndale R. W., Murphy G., Enghild J. J., Hohenester E., Nagase H. (2012). Structural insights into triple-helical collagen cleavage by matrix metalloproteinase 1. Proc. Natl. Acad. Sci. U.S.A..

[41] Pejler G., Lunderius C., Tomasini-Johansson B. (2000). Macrophages synthesize factor X and secrete factor X/Xa-containing prothrombinase activity into the surrounding medium. Thromb. Haemost..

[42] Busso N., Hamilton J. A. (2002). Extravascular coagulation and the plasminogen activator/plasmin system in rheumatoid arthritis. Arthritis Rheum..

[43] Vidal B., Serrano A. L., Tjwa M., Suelves M., Ardite E., De Mori R., Baeza-Raja B., Martinez de Lagran M., Lafuste P., Ruiz-Bonilla V. (2008). Fibrinogen drives dystrophic muscle fibrosis via a TGFβ/alternative macrophage activation pathway. Genes Dev..

